# Mental health and post-traumatic stress disorder in firefighters: an integrated analysis from an action research study

**DOI:** 10.3389/fpsyg.2023.1259388

**Published:** 2023-10-27

**Authors:** Joana Oliveira, Joana Aires Dias, Isabel Catarina Duarte, Salomé Caldeira, António Reis Marques, Vítor Rodrigues, João Redondo, Miguel Castelo-Branco

**Affiliations:** ^1^Coimbra Institute for Biomedical Imaging and Translational Research (CIBIT), Institute for Nuclear Sciences Applied to Health (ICNAS), University of Coimbra, Coimbra, Portugal; ^2^Faculty of Medicine (FMUC), University of Coimbra, Coimbra, Portugal; ^3^Centre for Prevention and Treatment of Psychological Trauma (CPTTP), Department of Psychiatry, Coimbra University Hospital Centre (CHUC), Coimbra, Portugal

**Keywords:** psychopathology, PTSD, firefighters, prevention, intervention, action-research

## Abstract

**Introduction:**

The presence of post-traumatic stress disorder **(**PTSD) symptomatology in firefighters is an ever-pressing issue that requires close attention for adequate interventions. The present study investigated PTSD and global psychopathology prevalence in a sample of highly risk-exposed Portuguese firefighters, collected after the widespread deadly wildfires in 2017 that ravaged the country. Following an action research approach, the aim of this study was to depict this sample and examine the impact of cumulative adverse experiences on their mental health, which is a phenomenon worth attention.

**Method:**

From an initial sample of 283 firefighters who manifested interest in participating, a total of 139 firefighters from the Coimbra District, of whom 130 unequivocally experienced a potentially traumatic/adverse event as a firefighter, completed BSI (to obtain indicators on psychopathology), QEPAT (an inventory of adverse events possibly experienced as a firefighter), and PCL-5 (a measure of PTSD symptomatology) through an online survey during the year 2018 by the Regional Medical Organization, as proposed and supervised by the local Centre for Prevention and Treatment of Psychological Trauma (CPTTP).

**Results:**

We found a global prevalence of 8.6% of possible PTSD and 14.4% of possible psychopathology (*n* = 139). When considering only firefighters who unequivocally reported a potentially traumatic/adverse event as a firefighter (*n* = 130), 9.2% present possible PTSD, and 13.8% present possible global psychopathology. This sample experienced a mean of 28 adverse events during firefighting work. Linear regressions (*n* = 118) demonstrated that the perceived severity of the most traumatic event reported and the experience of more adverse events were both related to an increase in PTSD symptomatology. Global psychopathology was associated with PCL-5 scores, with an emphasis on paranoid ideation, hostility, depression, anxiety, and phobic anxiety.

**Discussion:**

The severe wildfires of 2017 did not impact PTSD scores in this sample (collected the year after), suggesting that cumulative adverse events are more important than particular episodes. However, the number of reported events was related to PTSD scores. These results can be used to develop interventions that target all firefighters by addressing risk and protective factors. This action research study motivated specialized aid for firefighters involved in this study.

## 1. Introduction

Firefighters are often exposed to multiple threats and demands, confronted with multiple adverse stimuli and critical hostile events, under uncontrollable and unexpected scenarios, and occasionally even facing catastrophic and dangerous environments. These workers are trained to act toward uncertain situations, which require immediate action to surpass and cope with potentially traumatic incidents. Long and continued exposure to chronic and acute stressors makes firefighters one of the most vulnerable and highest-risk groups among emergency workers (Pinto et al., 2015; Onyedire et al., 2017). Portuguese firefighters are a particularly at-risk group due to the extensive fires and wildfires that take place every year in Portugal. Indeed, the impact of being a firefighter was recently highlighted by the Portuguese Council of Psychologists (Ordem dos Psicólogos Portugueses, 2022).

The most common negative mental health outcomes found in this population include emotional exhaustion (Afonso et al., 2019), sleep issues (Fullerton et al., 1992; Haslam and Mallon, 2003; Harvey et al., 2016; Frost et al., 2021), diminished attention, alertness, and cognitive performance (Sindhu et al., 2020), social dysfunction (Wagner et al., 1998), substance abuse (Marcelino et al., 2012; Harvey et al., 2016; Sindhu et al., 2020), anxiety (Sindhu et al., 2020; Wagner et al., 2021b), depression (Harvey et al., 2016; Salleh et al., 2020; Sindhu et al., 2020; Wagner et al., 2021b), and other psychopathological symptoms (Heinrichs et al., 2005; Wagner et al., 2010, 2021a,b). Related complications include intrusive memories (Fullerton et al., 1992; Clohessy and Ehlers, 1999; Vara and Queirós, 2018), suicidal ideation (Carpenter et al., 2015), suicidal behaviors (Stanley et al., 2015), and stress-related issues, including symptoms of post-traumatic stress disorder (PTSD) (e.g., Del Ben et al., 2006; Carvalho and Maia, 2009b; Berger et al., 2012; Salleh et al., 2020; Sindhu et al., 2020) and peritraumatic symptoms (Marcelino et al., 2012). Biologically associated problems include physical complaints (Fullerton et al., 1992; Carvalho and Maia, 2009a; Marcelino et al., 2012), psychosomatic manifestations (Wagner et al., 1998), and elevated risk for cardiovascular events, diabetes, or altered immune response (Sindhu et al., 2020). In this regard, the study conducted by Marmar et al. (1996) emphasized the importance of studying the psychobiological effects of daily operations in addition to large-scale, critical events. In a systematic review, the mental health profile of first responders was presented, including 18 studies with firefighters (see Jones, 2017).

Regarding levels of PTSD, overall, the existing literature provides evidence for higher levels of this disorder among firefighters compared to the general population, despite the heterogeneity observed in studies, as also noted by Becker et al. (2022). A systematic review (Wagner et al., 2021a) estimated that firefighters exposed to routine daily adverse incidents presented a mean prevalence of probable PTSD of 14.3% (with a large range between 3.9 and 54%), whereas the Canadian general population showed a prevalence of 3.5% (1-year rate) and 8% (lifetime rate). The same evidence was found for firefighters after involvement in large-scale events (mean estimated prevalence of 12.3%) (Wagner et al., 2021b). Evidence from other studies found prevalence rates of PTSD in firefighters between 1.9 and 57% (in contrast to 3.72–37.8% in the military workforce—for a review see Obuobi-Donkor et al., 2022), percentages between 6 and 48% (Vara et al., 2021), and Langtry et al. (2021) cited 5–22%, while values in the general population from various countries vary between 1.3 and 3.5%, in contrast with the 10% prevalence found in rescue workers (Berger et al., 2012). Using the gold standard for measuring PTSD [Portuguese version of this instrument was develop by R. Ferreira, L. Ribeiro, P. Santos, and A. Maia in 2016 (Silva, 2018); the PTSD Checklist for DSM-5–PCL-5 scale (Weathers et al., 2013)], Chiang et al. (2021) found 21% with a probable diagnosis among a total of 164 professional firefighters, whereas Sun et al. (2020) found 4.9% (*n* = 409) and Shi et al. (2021) found 1.9% (*n* = 261) both within Chinese samples. Ramos (2019) noted a prevalence of 2% for PTSD and 21% for peritraumatic dissociative symptoms among 736 Chilean volunteer firefighters. Koenen et al. (2017) analyzed data from 24 countries (WHO, World Mental Health Surveys), with a total of 71,083 adults. They found a lifetime prevalence of PTSD of 3.9% in the total sample and 5.6% among the trauma-exposed individuals, with variations across countries (values for all countries combined).

In Portugal, Marcelino and Gonçalves (2012) reported that among a sample of 830 firefighters from volunteer corporations, 8% met the diagnosis of PTSD, as measured by the Posttraumatic Stress Disorder Checklist—Civilian Version (PCL-C; original version: Weathers et al., 1993; Portuguese version: Marcelino and Gonçalves, 2012). In another study (Pinto et al., 2015), resorting to the Response to the Traumatic Event Scale (McIntyre and Ventura, 1996), it was found that ~12% of firefighters (from 28 northern departments) met the probable criteria. Using the PCL-5, Lima et al. (2016) identified that among 95 firefighters and municipal police workers, 10% had PTSD symptoms. Using the same scale, Mesquita (2018) found that 5.9% of 664 firefighters from 18 districts on the Portuguese mainland met the provisional diagnosis of PTSD. On the other hand, the prevalence for the general Portuguese population was close to 8%, as concluded in the first Portuguese epidemiological study of PTSD (Albuquerque et al., 2003). Later, the results from the first national epidemiological report of mental health (Almeida and Xavier, 2013) showed that Portugal represents the second-greatest prevalence of lifetime PTSD (5.3%) among the 14 high-income European countries participating in the survey, preceded by Northern Ireland (Koenen et al., 2017).

To the best of our knowledge, our study is the first to evaluate PTSD symptomatology (using the gold standard, PCL-5) and psychopathology in firefighters after the extreme and fatal wildfires of 2017 that claimed the lives of 117 individuals (San-Miguel-Ayanz et al., 2020) and ravaged more than 50 thousand hectares in Portugal (Viegas, 2018). As pointed out by Leone et al. (2023), “these type of questions (relating to behaviors similar to PTSD symptoms and psychological support, following the wildfires of 2017) have also never been examined for Portugal” (p. 16). Despite the large body of literature on the effects of trauma and stress among firefighters, there is still a gap in knowledge regarding their mental health and implications for family members, as well as other medium- and long-term consequences, such as compromised decision-making, inability to manage situations, task avoidance, and earlier retirement.

With regard to psychopathology and emotional distress, Carvalho and Maia (2009a) reported that ~17% of the total sample of Portuguese firefighters (*n* = 296) reported symptomology compatible with psychopathological disorders, as measured by the Brief Symptom Inventory (BSI; Derogatis, 1993; Portuguese version: Canavarro, 1999). Using the same measure, Pinto et al. (2015) reported that close to 19% of their firefighter sample (*n* = 397) surpassed the cutoff point indicative of probable emotional pathology (PSDI global score ≥1.7). Additionally, Teoh et al. (2019) detected 13% of psychiatric morbidity within a sample of 312 Brazilian firefighters. Wagner et al. (2010) found higher levels of anxiety, interpersonal sensitivity, psychoticism, and hostility within a sample of 94 paid-professional firefighters as measured by the Symptom Checklist 90–Revised (SCL-90-R, original version: Derogatis, 1983) when compared with 91 non-emergency service workers.

Portugal is devastated by forest fires every year, notwithstanding other types of fires, which have severe economic repercussions and social impacts. The main purpose of this action research study was to examine the mental health of firefighters and to investigate sociodemographic and other variables that contribute as risk or protective factors for mental health among a sample of Portuguese firefighters, including responses to larger events but also to daily incidents, analyzing the impact of cumulative exposure. In other words, we aimed to determine relevant variables underlying their mental health issues. We also hope to raise public awareness, including firefighters and their families, to seek earlier and continued specialized support and help to sensitize healthcare systems and other associations of firefighters to invest in prevention and early diagnosis programs (e.g., primary healthcare or mental healthcare services, namely, the CPTTP response). The focus is on resilience abilities (Onyedire et al., 2017), social support and healthcare, and anticipating psychological damage in firefighters.

## 2. Materials and methods

### 2.1. Participants

The initial sample of potentially interested participants was composed of 283 volunteers and professional firefighters from volunteer corporations of firefighters in the District of Coimbra. Although this sample was collected only from corporations from this district, two essential aspects underpin this methodology—on the one hand, the action research purpose of the study anticipates specialized support for involved firefighters, so the sample collection was limited to the radius of action of the clinical team; on the other hand, the 2017 extreme fires significantly affected the Central region of Portugal, with two fire outbreaks in two zones south-east of Coimbra.

**Figure 1:**
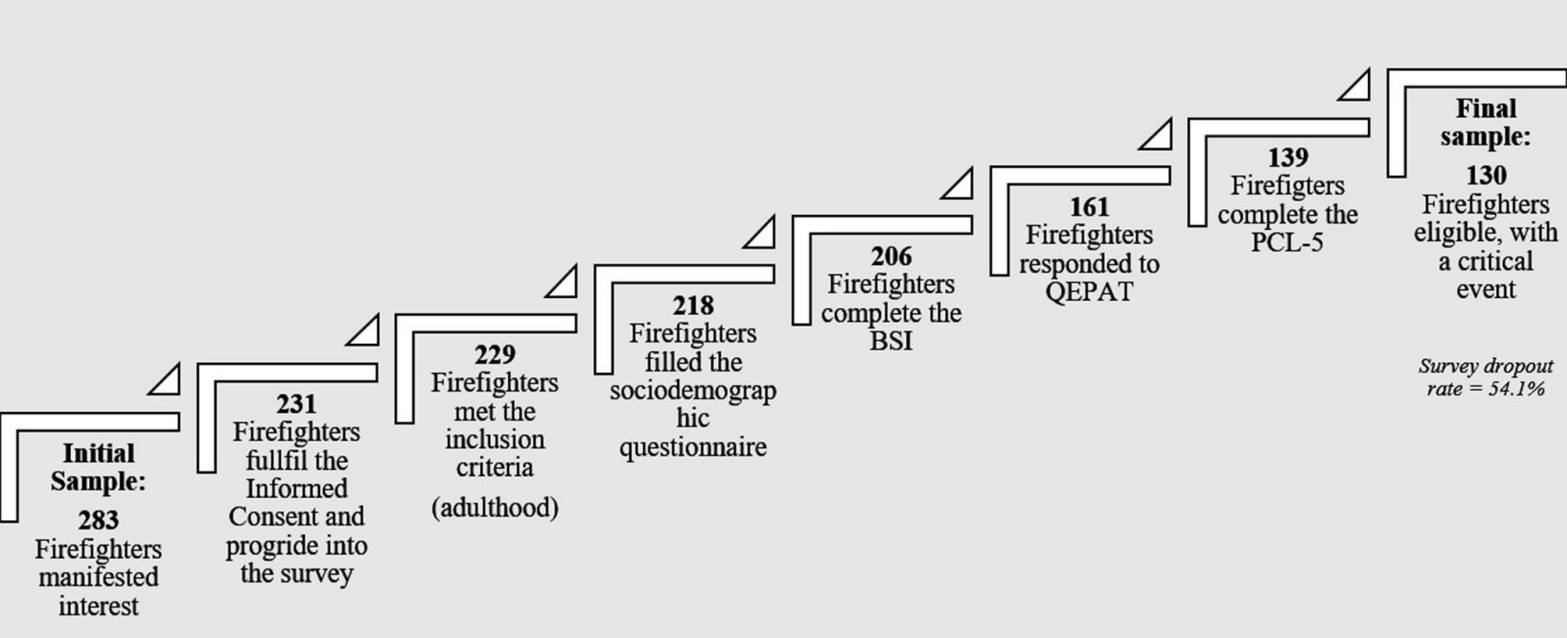
Participant workflow: an overview of the participants' progression in the study and survey dropout rate. BSI, Brief Symptom Inventory; QEPAT, *Questionário de Exposição e Perturbação dos Acontecimentos Traumáticos*, a questionnaire related to the exposure and disturbance of traumatic events; PCL-5, PTSD Checklist for DSM-5.

The survey dropout rate increased incrementally as participants progressed through the reporting of measures. Indeed, 52 participants did not provide signed consent for participation or did not respond after signing the informed consent, and 2 participants were excluded because they were underage (<18 years old). Furthermore, 11 participants did not complete the sociodemographic questionnaire, 12 did not complete the *Brief Symptom Inventory* questionnaire (BSI), 45 did not complete a questionnaire related to the exposure and disturbance of potentially traumatic events [in Portuguese: *Questionário de Exposição e Perturbação dos Acontecimentos Traumáticos* (QEPAT)], and 22 did not complete the *PTSD Checklist for DSM-5* (PCL-5). The aforementioned participants were excluded for failing to complete all measures. This yielded a sample of 139 participants. Finally, a selection criterion for participation in the study included the participants who had experienced a traumatic/adverse event as a risk factor, considering that the instrument that evaluates post-traumatic stress symptoms (PCL-5) does so in light of a reported traumatic experience. Participants who reported never having experienced such an event and participants who did not provide a clear response (*n* = 9) were considered when calculating prevalence rates (*n* total = 139) but excluded from the remaining analyses. This rendered a final sample of 130 Portuguese firefighters, with a dropout/exclusion rate of 54.1%. This rate could be related to the extension of the procedure (a wide online survey) and the nature and length of the study (see the Participant workflow, Figure 1).

### 2.2. Instruments

The participants completed a voluntary and anonymized online survey that included translated/adapted/validated self-report questionnaires selected by the expert CPTTP team, which included questions about stress, adverse events, and the impact on firefighters' health, in order to delineate more accurate prevention/intervention programs in the healthcare system.

The survey lasted ~30 min; duplicate responses were not allowed, and participants could cease the questionnaire at any time. The order of presentation was as follows:

Sociodemographic and clinical data were collected using a form with sociodemographic and firefighting activity-related questions (in Portuguese, the *Questionário Sociodemográfico e da atividade do bombeiro*; Gently provided by Lopes R., Mendes J. M. and Maia A.; see acknowledgments). This questionnaire includes items on gender, age, education, marital status, number of children, consumption habits (alcohol, coffee, tobacco, medication), employment status, if they are on a voluntary or professional regimen, years in active firefighting service, number of hours worked per week, and specific occupation as a firefighter.

The Brief Symptom Inventory (BSI, Derogatis, 1993; Portuguese version: Canavarro, 1999) assesses psychopathological symptoms, comprising nine primary dimensions of symptomatology (Somatization, Obsessive-Compulsive, Interpersonal Sensitivity, Depression, Anxiety, Hostility, Phobic Anxiety, Paranoid Ideation, and Psychoticism) and three global indexes of distress [general severity index (GSI), positive symptom distress index (PSDI), positive symptom total (PST)] as indicators of emotional disorder. We used the original terms adopted by the authors (Derogatis and Melisaratos, 1983). It is a self-report questionnaire, composed of 53 items, in which participants rate the extent of disturbance for each symptom during the past week using a 5-point Likert scale that spans from (0) “not at all” to (4) “extremely.” According to the author (Canavarro, 2007), the PSDI is the best indicator to measure psychosymptomatology as it is the index that has achieved the highest discrimination scores. The possible minimum for this indicator is 0, and the maximum is 4, with values equal to or higher than 1.7 indicating possible psychopathology.

Internal consistency for BSI subscales in the current study was acceptable (the large majority of subscales obtained an alpha >0.74; only the phobic anxiety dimension presented an alpha considered low of 0.64). The Portuguese version of the instrument found similar patterns of consistency [Phobic Anxiety and Psychoticism presented the lowest values (α = 0.62; Canavarro, 2007)].

Participants also completed a Portuguese questionnaire with a list of 43 potentially traumatic/adverse events directly related to firefighting work, which measures the frequency of exposure and the firefighter's perception of disturbance for each event [in Portuguese: *Questionário de Exposição e Perturbação dos Acontecimentos Traumáticos* (QEPAT) Carvalho and Maia, 2009a]. Items are classified on a 5-point scale that ranges from (0) “never” to (4) “frequently” (frequency of exposition subscale) and (0) “not at all” to (4) “very much” (perceived disturbance subscale). The last item provides the respondent with the possibility to select and describe another event not listed and then classify it. The second part of the QEPAT includes the selection of the most disturbing (adverse, traumatic) event experienced by a firefighter, and participants were asked to provide a brief description of the event. Then, a classification of the traumatic degree of the experience was required on a 7-point scale ranging from “not traumatic” to “extremely traumatic.” Additional information was required, namely, the time elapsed since the episode occurred, experience as a victim or witness, severity of physical and psychological injuries as a result of the event, and finally, if the firefighter had already experienced a similar event.

In this sample, both the frequency scale (α = 0.94) and the disturbance scale (α = 0.97) of QEPAT obtained strong internal consistency. When applying the QEPAT (Spanish version, 37 items) to a sample of Chilean firefighters, the author (Ramos, 2019) found a Cronbach's alpha of 0.90 for the frequency scale. A similar result was found by Carvalho and Maia (2009a) using the first version of the questionnaire (40 items) (α = 0.93, disturbance subscale).

The PTSD Checklist for DSM-5 (Weathers et al., 2013; Portuguese study: Silva, 2018) is a 20-item self-report measure used to assess the severity of symptoms of post-traumatic stress disorder in the last month according to the DSM-5 criteria (American Psychiatric Association, 2013). Respondents indicated the frequency of each symptom, rated on a 0–4 scale, between “not at all” and “extremely,” with the scale having a minimum of 0 and a maximum of 80. The scale includes the four DSM-5 symptoms clusters: Intrusion (cluster B), Avoidance (cluster C), Negative Alterations in Cognition and Mood (NACM, cluster D), and Alterations in Arousal and Reactivity (AAR, cluster E). In this study, the version of PCL-5 without criterion A was used because the trauma/adverse event exposure is measured by the QEPAT. The participants completed the PCL-5 immediately after filling out the QEPAT, where they indicated the most critical experience during the firefighting activity. They then responded to the PCL-5 with respect to the event previously reported. In this regard, it should be noted that our intent was neither establishing a clinical diagnosis of PTSD nor performing a comprehensive evaluation. In order to achieve the study's aim and reach more extensive conclusions, we decided not to follow the exact definition of a traumatic event, as prescribed by criterion A of the DSM-5 and essential for the clinical diagnostic eligibility of PTSD. Rather, we included all participants who reported experiencing an unambiguous critical event as a firefighter and who stated a clarification of their adverse experience. In this regard, Stein et al. (2016) emphasized the singular nature of stressful, traumatic experiences, discussing their nosological and scientific implications.

The recommended cutoff interval for detection of probable PTSD in PCL-5 is between 31 and 33 (range for total score: 0–80) (National Center for PTSD, 2018). In this study, the lowest value was chosen as a threshold. Additionally, screening for a provisional diagnostic could be made by considering each item reported by the participant as “moderately” (rated with 2 points or higher). The level of internal consistency of the full-scale score in the current study was 0.94, as measured by Cronbach's alpha. In the study of Portuguese validation within a sample of firefighters, the values of Cronbach's alphas were 0.88 for the total score, 0.77 for Intrusion, 0.86 for Avoidance, 0.83 for NACM, and 0.94 for AAR (Silva, 2018).

### 2.3. Procedure

Data were collected by a multidisciplinary clinical group led by João Redondo, MD, coordinator of the Centre for Prevention and Treatment of Psychological Trauma (CPTTP), belonging to the Department of Psychiatry of the Coimbra University Hospital Centre (CHUC), a team with considerable expertise in stress- and trauma-related disorders and specifically interested in these topics.

The sample was collected *via* an online survey during the year 2018 (after the extensive forest fires that devastated Portugal in 2017, which claimed numerous human and animal lives and burned thousands of hectares and assets). The survey was hosted on a server from the Ordem dos Médicos (the Regional Council of Medical Doctors), which was made available to volunteer corporations of firefighters from the District of Coimbra with the support of the Federation of Firefighters from the District of Coimbra. This collaboration included a previous session at the National Fire Service School—Portugal (pole of Lousã), conducted by the senior psychiatrist (JR) among the various firefighters' leaders, in order to disseminate and explain the aims of the study, namely, the preventive facet of the project.

This action research study received the collaboration of the CIBIT Research Institute, ICNAS, and the Biostatistics and Epidemiology Departments, Faculty of Medicine of the University of Coimbra, and received support from the Ordem dos Médicos Portugueses (Centre Regional Office), the entity that serves as the regulatory body for physicians, with data entry as proposed and supervised by the CPTTP team.

The study has been approved by all relevant institutional review boards (local Ethics Committee of the Administração Regional de Saúde do Centro, ARS Centro). The procedures of the study were in accordance with the World Medical Association's Declaration of Helsinki. This study was inserted into a larger project with a preventive intervention view and has the ultimate purpose of outlining programs that enable firefighters to better cope and manage their response to adverse events/stress and of bringing awareness to this population. Specialized mental aid was made available for firefighters at the Centre for Prevention and Treatment of Psychological Trauma (CPTTP), belonging to the Department of Psychiatry of the Coimbra University Hospital Centre, in the psychiatric and psychotherapeutic areas.

## 3. Results

### 3.1. Sociodemographic characteristics, consumption habits, and firefighting activity

The final sample (*n* = 130) was composed of 69.2% male and 28.5% female respondents, with 2.3% choosing not to disclose this information. The mean age was 35.6 years old (*SD* = 10.1, *Mdn* = 36.0), with the respondents overall ranging between 19 and 58 years old. Approximately half (44.6%) of the sample completed the Portuguese secondary education (i.e., the current mandatory education in Portugal); 26.1% had less than secondary education, while 29.2% had an education level higher than secondary (of which 22.3% held a higher education degree). The majority of participants were integrated, simultaneously, into urban and rural corporations (56.2%). Regarding their household, a little less than half of the participants were not married (46.9%), while 50% were either married (35.4%) or in a non-marital relationship (14.6%). Close to half of the participants had children (47.7%), with most having only one child (54.8%). The sociodemographic data were collected using a Portuguese questionnaire (developed by Lopes, R., Mendes, J. M. and Maia, A., in 2016) to collect variables directly related to firefighting activity (in Portuguese, *Questionário Sociodemográfico e da atividade do bombeiro*). All the detailed sociodemographic characterizations of the participants can be found in Supplementary Table 1.

Regarding their consumption habits, ~40% of participants reported drinking alcohol regularly, while 2.3% openly opted not to respond. Although none of the participants considered that they had a drinking problem, two (1.5%) reported that their family members perceived they did. The vast majority (91.5%) reported drinking coffee on a regular basis. Concerning smoking habits, ~37% of participants reported smoking regularly. Finally, 21.5% of participants reported taking medication to treat depression, anxiety, sleeping disorders, or other related issues, with 5.4% taking these pharmacological aids on a daily basis.

Regarding their professional occupation, 78.5% of firefighters were employed, of which 81.5% were service providers (secondary sector).

The total sample averaged 13.8 years in active firefighting service (*SD* = 9.8, *Mdn* = 12.0). In relation to the firefighting activity, the majority of participants were integrated into a voluntary regimen (63.1%) or were in a hybrid regime (voluntary and professional) (26.2%). Considering the subsample of professional firefighters (including those who simultaneously work as volunteers), 37.5% were drivers, for a mean of 8.7 years (*SD* = 8.2, *Mdn* = 7.0), and approximately one-fifth (22.9%) was a member of a Permanent Intervention Team [PIT, in Portuguese *Equipas de Intervenção Permanente* (EIP)] that engages actively in firefighting activities in the scope of the Portuguese Civil Protection System, having held this occupation for a mean of 6.4 years (*SD* = 4.7, *Mdn* = 5.0). Members of PITs were the firefighters who reported working more hours weekly (*M* = 59.9, *SD* = 8.9, *Mdn* = 60.0).

All detailed sociodemographic characterizations, consumption habits, and characterizations of firefighting activities of the sample can be found in Supplementary Table 1.

### 3.2. Analysis of psychological and traumatic event measures

#### 3.2.1. Psychopathology

To grasp the emotional symptomatology of the sample and detect psychopathology, the three global indexes of the BSI and dimensions were analyzed. A cutoff value of PSDI ≥1.7 was used to determine emotional pathology, as recommended by the author (Canavarro, 2007). The mean score for the final sample (*n* = 130) was 1.3 (*SD* = 0.3, *Mdn* = 1.2). Although the sample mean does not surpass the threshold, 18 participants (13.8%) had values equal to or higher than the cutoff value of 1.7 in the PSDI, which can be indicative of psychopathology. When including the 9 participants who reported not having a traumatic event (*n* = 139), 2 additional participants surpassed the 1.7 cutoff (equal or higher), resulting in 20 (14.4%) with possible psychopathology.

Cutoff points were also calculated for all the subscales of the BSI to conduct an exploratory analysis, following Fisher's formula (Angoff, 1971) and according to the data collected by the author (Canavarro, 2007). Cutoff points were obtained by calculating [(*M*1 + *SD*1) + (*M*2 – *SD*2)]/2, where *M*1 and *SD*1 refer to the mean and standard deviation of emotionally disturbed individuals, and *M*2 and *Sd*2 refer to the mean and standard deviation of individuals from the general population. The data pertain to the normative Portuguese sample acquired by the author (Canavarro, 2007). Cutoff values for the subscales and the number of participants who surpass the threshold (equal or higher) are as follows: Somatization: 0.9 (*n* = 17); Obsessive-Compulsive: 1.6 (*n* = 17); Interpersonal Sensitivity: 1.1 (*n* = 34); Depression: 1.2 (n = 30); Anxiety: 1.3 (*n* = 16); Hostility: 1.1 (*n* = 28); Phobic Anxiety: 0.6 (*n* = 23); Paranoid Ideation: 1.3 (*n* = 50); Psychoticism: 0.9 (*n* = 26). Six participants surpassed the threshold for all subscales. These thresholds are only an informative detail and provide a comparison with the PSDI global score; they are not intended for clinical diagnosis capacity, although they provide screening value.

Considering that the Permanent Intervention Team (PIT) members perform active firefighting duties, it could be possible that the psychopathological profile of this subsample would be different from the total sample. For that reason, analyses were performed to detect psychopathology within this subgroup, as measured by the BSI questionnaire. Of the 11 PIT members, none had values surpassing the cutoff point (PSDI ≥ 1.7). One PIT member had values higher than the cutoff for the Hostility and Psychoticism dimensions, calculated within the scope of this study. Two other participants surpassed the cutoff values, one for the Paranoid Ideation subscale and the other for Psychoticism.

#### 3.2.2. Traumatic/adverse events during the firefighting activity

All the events listed in the QEPAT were experienced by our firefighting sample. However, only one participant (a PIT member) reported having experienced every event at least once. The sample experienced a mean of 27.7 events (*SD* = 9.1, *Mdn* = 29).

Regarding the three most frequently experienced events, the most frequently experienced event among the mean sample was being frequently involved in firefighting events where property and goods were at risk of burning (*M* = 2.5, *SD* = 1.1, *Mdn* = 3.0, range: 0–4), with 17 (13.1%) participants selecting the highest end-point of the scale (i.e., “frequently”), followed by 56 (43.1%) firefighters who selected the second highest end-point of the scale on this question, reporting having been involved in this type of event many times. The second most frequent event to which the sample was exposed was helping the elderly that were injured or at risk of injury and fragility (*M* = 2.3, *SD* = 1.1, *Mdn* = 2.0, range: 0–4), with 14 (10.8%) participants reporting having experienced this scenario frequently. Finally, the mean sample reported participating frequently in major fires or wildfires, or fires or wildfires that spanned over a long period of time more frequently (*M* = 2.1, *SD* = 1.0, *Mdn* = 2.0, range: 0–4), with six (4.6%) participants reported participating frequently in this event.

The participants were also questioned on the severity of the disturbance caused by each event. Even though most participants did not experience a specific event, many of them reported a certain level of disturbance regarding that type of event. For instance, of the 104 participants who reported never seeing or holding children's cadavers, 47 reported some degree of disturbance due to the event, even though they themselves did not experience it.

The one event that had the highest mean score of severity was witnessing a colleague's death or severe injury while in active service (*M* = 2.9, *SD* = 1.2, *Mdn* = 3.0), followed by hearing radio communications about firefighter colleagues who were in danger, injured, or deceased (*M* = 2.9, *SD* = 1.1, *Mdn* = 3.0) and having to rescue a colleague firefighter at risk of death or severely injured while in active service (*M* = 2.8, *SD* = 1.2, *Mdn* = 3.0). The participants who reported never having experienced specific events were not included in the analyses of the disturbing impact of those events.

The participants then stated which event, of all events experienced, they considered to be the most significant and were required to briefly describe it. Afterwards, they reported how traumatic they considered that event to be on a self-reported 7-point Likert scale, resulting in a mean of 4.5 points (*SD* = 1.6, *Mdn* = 5.0). The majority of participants witnessed the event (*n* = 87, 66.9%), with 43 (33.1%) being victims, and 53.1% of participants were referring to an event that happened 1 year before (*M* = 4.3, *SD* = 6.6, *Mdn* = 1.0). In all, 15 participants reported having had physical injuries due to the event (1 with extremely severe injuries), and 32 reported psychological repercussions (none reporting severe consequences). It is noteworthy that of the 87 participants who witnessed the event, 4 reported having suffered physical injuries. Moreover, 17 (13.1%) participants had already experienced a similar event before the one they selected as the most traumatic.

#### 3.2.3. PTSD symptoms and severity

Considering the selected event from the QEPAT to complete the PCL-5, in regard to the participants' most significant event experienced during the firefighting service, eight participants surpassed the threshold of the PTSD indicator of 31 (*Min* = 33.0, *Max* = 50.0). When following the DSM-5 diagnostic rule for PTSD—at least one symptom of Criteria B and C and two symptoms of Criteria D and E, reported by the participant as being “moderately” endorsed or higher (American Psychiatric Association, 2013), 10 participants fulfilled the criteria for a provisional diagnosis of PTSD, one being a PIT member. Six participants surpassed the value of 31 on the PCL-5 full scale and fulfilled the DSM-5 criteria for PTSD. In total, only 12 participants fulfilled either one or both criteria, both when considering the total of 139 participants who filled out the PCL-5 (8.6% who fulfilled possible PTSD criteria) or the 130 who reported having experienced a traumatic event (9.2% who fulfilled possible PTSD criteria). The prevalence is stated here considering the larger sample (8.6%), although further analysis has been conducted considering solely the participants who unequivocally reported having experienced a traumatic event, as previously stated. The participants who fulfilled PTSD criteria in the final sample (*n* = 130) will be treated as an independent subsample, as it does not represent the majority of the firefighter sample but should not be neglected due to the higher prevalence of PTSD in firefighters (e.g., Pinto et al., 2015; Mesquita, 2018). However, some caution is needed when interpreting these findings due to the different sizes of each subsample. A binominal test indicated that the prevalence of PTSD in the sample of the present study (*n* = 139, 8.6%) is not different from that found in Mesquita (2018) (5.9%; *exact-p* = 0.120). This remains true when considering the final sample of 130 (9.2%, *exact-p* = 0.083).

Concerning the number of events experienced, as measured by the QEPAT, the first subsample that did not surpass the PTSD (*n* = 118) thresholds reported having experienced a mean of 26.5 events (*SD* = 9.2, *Mdn* = 28.5), with the most traumatic event having a mean of 4.3 points out of 7 (*SD* = 1.6, *Mdn* = 5.0). The latter subsample, which did surpass the thresholds, experienced a mean of 34 events (*SD* = 5.8, *Mdn* = 35.0), with the most traumatic event having a mean of 6.5 points out of 7 (*SD* = 0.7) and a median of 7.0, with more than half (*n* = 7, 58.3%) choosing the highest point of the scale. Approximately the same percentage of participants in the possible PTSD subsample (33.3%) and in the non-PTSD subsample (33.1%) reported having been a victim of the event they described as most traumatic. A total of 11.9% (*n* = 14) of the non-PTSD subsample reported having suffered physical injuries due to the event, while only one participant (8.3%) of the possible PTSD subsample reported being physically injured in the event, additionally indicating that they had been severely injured.

It should also be noted that three (25%) of the 12 participants who fulfilled one of the PTSD criteria reported not having psychological repercussions as a consequence of the event. On the other hand, 23 (19.5%) of participants from the non-PTSD subsample reported having psychological issues as a result of experiencing the event. In all, 2 (16.7%) participants in the possible PTSD subsample had already experienced a similar event, with 15 in the non-PTSD subsample (12.7%) having had a previous experience.

The complete descriptive statistics and Cronbach's alpha of the Global Indexes and subscales of the BSI and PCL-5 for the sample that did not fulfill any of the PTSD criteria can be found in Table 1. Statistics for the subsample that fulfilled the PTSD criteria can be found in Table 2. The non-PTSD sub's alpha values showed adequate internal consistency across all subscales (min = 0.72, max = 0.89), with the exception of the Phobic Anxiety BSI subscale, which yielded a low value (α = 0.50). This indicates that scores in this subscale may be unreliable in representing participants' phobic anxiety. A similar result was found by the author (Canavarro, 2007) for this scale. In the PTSD subsample, Cronbach's alpha ranged from very poor to excellent (min = 0.41, max = 0.92).

**Table 1:** Descriptive statistics for BSI and PCL-5 (Firefighters' sample that did not fulfill probable PTSD criteria).

**Scales and subscales**		**Statistics**				
		***M***	***SD***	***Mdn***	***Min–Max***	α
**Brief symptom inventory (BSI)**						
	Positive symptom distress index (PSDI)	1.3	0.4	1.2	0.0–2.9	
	Positive symptom total (PST)	21.5	13.5	21.5	0.0–49.0	
	General severity index (GSI)	0.6	0.5	0.5	0.0–2.3	
	Somatization	0.4	0.4	0.2	0.0–2.0	0.77
	Obsessive-compulsive	0.8	0.6	0.7	0.0–3.5	0.85
	Interpersonal sensitivity	0.7	0.7	0.5	0.0–3.5	0.85
	Depression	0.7	0.7	0.5	0.0–3.3	0.89
	Anxiety	0.5	0.6	0.3	0.0–3.7	0.85
	Hostility	0.6	0.6	0.4	0.0–3.0	0.84
	Phobic anxiety	0.2	0.3	0.0	0.0–1.6	0.50
	Paranoid ideation	1.0	0.7	0.8	0.0–3.2	0.85
	Psychoticism	0.4	0.5	0.2	0.0–2.4	0.72
**PTSD checklist for DSM-5 (PCL-5)**		7.2	7.0	5.0	0.0–29.0	0.88
	Intrusion symptoms	2.1	2.5	1.0	0.0–12.0	0.78
	Avoidance	1.1	1.3	0.5	0.0–6.0	0.72
	NACM	1.5	2.2	0.0	0.0–9.0	0.76
	AAR	2.5	2.7	2.0	0.0–11.0	0.74

NACM, Negative Alterations in Cognition and Mood; AAR, Alterations in Arousal and Reactivity.

**Table 2:** Descriptive statistics for BSI and PCL-5 (Firefighters' sample that did fulfill probable PTSD criteria).

**Scales and subscales**		**Statistics**				
		***M***	***SD***	***Mdn***	***Min*** **–** ***Max***	α
**Brief symptom inventory (BSI)**						
	Positive symptom distress index (PSDI)	1.7	0.3	1.7	1.2–2.2	
	Positive symptom total (PST)	40.1	9.2	42.5	25.0–50.0	
	General severity index (GSI)	1.3	0.5	1.4	0.7–2.0	
	Somatization	0.9	0.6	0.8	0.3–2.0	0.90
	Obsessive-compulsive	1.5	0.6	1.5	0.7–2.5	0.82
	Interpersonal sensitivity	1.5	0.7	1.5	0.3–3.0	0.80
	Depression	1.6	0.5	1.7	0.7–2.2	0.68
	Anxiety	1.3	0.6	1.3	0.5–2.2	0.83
	Hostility	1.5	0.7	1.6	0.2–2.8	0.82
	Phobic anxiety	0.7	0.6	0.8	0.0–1.8	0.70
	Paranoid ideation	2.0	0.6	2.2	0.8–2.6	0.51
	Psychoticism	1.1	0.5	1.0	0.2–1.8	0.64
**PTSD checklist for DSM-5 (PCL-5)**		34.1	6.9	33.5	26.0–50.0	0.62
	Intrusion symptoms	9.8	3.5	10.0	5.0–15.0	0.82
	Avoidance	4.4	1.8	4.0	1.0–8.0	0.92
	NACM	10.5	3.6	9.0	5.0–17.0	0.41
	AAR	9.4	2.1	9.5	7.0–13.0	−0.23

NACM, Negative Alterations in Cognition and Mood; AAR, Alterations in Arousal and Reactivity.

Correlations between the general scores of both scales and subscales for both PTSD and non-PTSD subsamples can be found in Supplementary Tables 2, 3. In the non-PTSD subsample, correlations were significant for all BSI subscales and the Positive Symptom Distress Index (PSDI) (min. *r* = 0.50, max. *r* = 0.74) and between all BSI subscales (min. *r* = 0.51, max. *r* = 0.82). The PCL-5 full scale correlated with all its subscales (min. *r* = 0.70, max. *r* = 0.86) and with BSI's PSDI (*r* = 0.33, *p* < 0.001) and BSI subscales (min. *r* = 0.23, max. *r* = 0.53). Significant correlations were also found between all PCL-5 subscales (min. *r* = 0.39, max. *r* = 0.74). The Positive Symptom Distress Index (PSDI) was significantly correlated with all PCL-5 subscales (min. *r* = 0.25, max. *r* = 0.28). Some correlations between PCL-5 subscales and BSI subscales were non-significant, with most being in regard to the Avoidance subscale, which is indicative of probable distinct constructs.

While most correlations in the non-PTSD subsample were significant, the majority of correlations in the PTSD subsample were non-significant. The only noteworthy correlations were in the Anxiety subscale in relation to Somatization (*r* = 0.92, *p* < 0.001) and to Obsessive-Compulsive (*r* = 0.93, *p* < 0.001) and between the PSDI global score and the Depression subscale (*r* = 0.92, *p* < 0.001). All significant correlations for the PTSD subsample can be found in Supplementary Table 3.

### 3.3. Inferential analysis

In the current study, the subsample of participants who surpassed the threshold of PTSD according to the PCL-5 or DSM-5 rule was too small (*n* = 12) to compare to the non-PTSD subsample (*n* = 118). Hence, the inferential analysis has been conducted solely for the non-PTSD subsample as it is the sample that best represents the majority of firefighters (Pinto et al., 2015), given that most firefighters do not have PTSD.

A first analysis was conducted using the BSI and PCL-5 global and specific scores to determine the influence of psychopathological symptoms on the level of PTSD. All assumptions for parametric analyses were tested, and, when not verified, the adequate non-parametric alternative was applied. Deviation from normality was tested according to the criteria proposed by Kline (2016). No significant deviation was found.

A simple linear regression (*Enter* method) showed that the BSI global score, measured by the Positive Symptom Distress Index (PSDI), influenced the PCL-5 full-scale score [β = 0.33, *t*(116) = 3.72, *p* < 0.001]. A similar pattern was found when using the Positive Symptom Total (PST) index [β = 0.52, *t*(116) = 6.51, *p* < 0.001] and the General Severity Index (GSI) [β = 0.47, *t*(116) = 5.66, *p* < 0.001]. The same pattern of results was found for each PCL-5 subscale (Table 3).

**Table 3:** Simple regression of BSI global scores on PCL-5 scale and subscales.

**BSI global score**	**PCL-5 full scale**				**PCL-5 subscales**															
					**Intrusion symptoms**				**Avoidance**				**NACM**				**AAR**			
	***R** ^2^*	β	***t***	***p***	***R** ^2^*	β	***t***	***p***	***R** ^2^*	β	***t***	***p***	***R** ^2^*	β	***t***	***p***	***R** ^2^*	β	***t***	***p***
PSDI	0.11	0.33	3.72	**< 0.001**	0.06	0.25	2.82	**0.006**	0.06	0.25	2.82	**0.006**	0.07	0.26	2.87	**0.005**	0.08	0.28	3.10	**0.002**
PST	0.27	0.52	6.51	**< 0.001**	0.12	0.34	3.94	**< 0.001**	0.06	0.25	2.75	**0.007**	0.26	0.51	6.29	**< 0.001**	0.24	0.49	6.02	**< 0.001**
GSI	0.22	0.47	5.66	**< 0.001**	0.11	0.33	3.71	**< 0.001**	0.07	0.26	2.87	**0.005**	0.20	0.44	5.34	**< 0.001**	0.17	0.41	4.89	**< 0.001**

PSDI, Positive Symptom Distress Index; PST, Positive Symptom Total; GSI, General Severity Index; PCL-5, PTSD Checklist for DSM-5; NACM, Negative Alterations in Cognition and Mood; AAR, Alterations in Arousal and Reactivity.

Bold values indicate significant effects.

To discern if any specific dimension of psychopathology has accentuated influence on the sample's PCL-5 scores, multiple linear regressions were conducted with all the BSI subscales as predictors and the PCL-5 full scale and subscales as outcome measures. The overall model explained 36% of the PCL-5 full-scale variance [adjusted *R*^2^ = 0.36, *F*_(9, 108)_ = 8.25, *p* < 0.001]. The dimensions that predicted the most variance in PCL-5 scores were the Paranoid Ideation [β = 0.40, *t*(108) = 2.99, *p* = 0.004] and Hostility subscales [β = 0.40, *t*(108) = 3.43, *p* < 0.001], followed by the Depression subscale [β = −0.35, *t*(108) = −2.19, *p* = 0.030], which later had a negative effect on PCL-5 scores. The Anxiety [β = 0.32, *t*(108) = 2.02, *p* = 0.046] and Phobic Anxiety subscales [β = −0.24, *t*(108) = −2.24, *p* = 0.027] also had a significant, albeit smaller, influence on the PCL-5 full scale scores, with the former having a positive and the latter a negative effect.

Not all the aforementioned dimensions of psychopathology influenced post-traumatic stress symptoms equally. For instance, while Depression also negatively affected alterations in mood and cognition (NACM) [β = −0.49, *t*(108) = −2.92, *p* = 0.004] and alterations in arousal and reactivity (AAR) [β = −0.37, *t*(108) = −2.09, *p* = 0.039], it did not affect Intrusion Symptoms [β = −0.01, *t*(108) = −0.06, *p* = 0.955] or Avoidance [β = −0.27, *t*(108) = −1.48, *p* = 0.142]. On the other hand, Hostility influenced Intrusion Symptoms [β = 0.52, *t*(108) = 4.05, *p* < 0.001] and Avoidance [β = 0.32, *t*(108) = 2.39, *p* = 0.019], and although it did not influence NACM [β = 0.23, *t*(108) = 1.84, *p* = 0.069] or AAR [β = 0.22, *t*(108) = 1.73, *p* = 0.087], a clear trend is noticeable since *p*-values were below the significance level of 0.10. Regarding the Anxiety and Phobic Anxiety subscale scores, the former predicted NACM [β = 0.46, *t*(108) = 2.77, *p* = 0.007], and the latter predicted Intrusion Symptoms [β = −0.34, *t*(108) = −2.93, *p* = 0.004]. Paranoid Ideation had an effect across all PCL-5 subscales, with the exception of NACM [β = 0.21, *t*(108) = 1.48, *p* = 0.141], which had no effect on alterations in cognition or mood. Finally, Somatization predicted Negative Alterations in Mood and Cognition (NAMC) [β = −0.26, *t*(108) = −2.08, *p* = 0.040], having no effect on neither the PCL-5 full-scale scores [β = −0.19, *t*(108) = −1.56, *p* = 0.123] nor its other subscales. All multiple linear regression scores can be found in Table 4.

**Table 4:** Multiple regression of BSI subscales on PCL-5 subscales.

**BSI subscales**	**PCL-5 full scale**			**PCL-5 subscales**											
				**Intrusion symptoms**			**Avoidance**			**NACM**			**AAR**		
	**Adjusted R**^2^ = **0.36**, *F*_(9, 108)_ = **8.25**, ***p**<* **0.001**			**Adjusted R**^2^ = **0.23**, *F*_(9, 108)_ = **4.94**, ***p**<* **0.001**			**Adjusted R**^2^ = **0.19**, *F*_(9, 108)_ = **3.97**, ***p**<* **0.001**			**Adjusted R**^2^ = **0.30**, *F*_(9, 108)_ = **6.47**, ***p**<* **0.001**			**Adjusted R**^2^ = **0.22**, *F*_(9, 108)_ = **4.70**, ***p**<* **0.001**		
	β	***t***	***p***	β	***t***	***p***	β	***t***	***p***	β	***t***	***p***	β	***t***	***p***
Somatization	−0.19	−1.56	0.123	−0.11	−0.82	0.415	−0.18	−1.35	0.179	–**0.26**	–**2.08**	**0.040**	−0.08	−0.61	0.542
Obsessive-compulsive	0.04	0.31	0.755	−0.04	−0.25	0.803	−0.09	−0.56	0.575	0.03	0.19	0.854	0.17	1.10	0.274
Interpersonal sensitivity	0.26	1.60	0.114	0.14	0.75	0.455	0.36	1.97	0.052	0.30	1.73	0.087	0.13	0.73	0.469
Depression	–**0.35**	–**2.19**	**0.030**	−0.01	−0.06	0.955	−0.27	−1.48	0.142	–**0.49**	–**2.92**	**0.004**	–**0.37**	–**2.09**	**0.039**
Anxiety	**0.32**	**2.02**	**0.046**	0.12	0.71	0.478	0.19	1.09	0.276	**0.46**	**2.77**	**0.007**	0.24	1.40	0.166
Hostility	**0.40**	**3.43**	**< 0.001**	**0.52**	**4.05**	**< 0.001**	**0.32**	**2.39**	**0.019**	0.23	1.84	0.069	0.22	1.73	0.087
Phobic anxiety	–**0.24**	–**2.24**	**0.027**	–**0.34**	–**2.93**	**0.004**	−0.21	−1.77	0.079	−0.08	−0.74	0.460	−0.13	−1.12	0.267
Paranoid Ideation	**0.40**	**2.99**	**0.004**	**0.33**	**2.20**	**0.030**	**0.38**	**2.46**	**0.015**	0.21	1.48	0.141	**0.39**	**2.60**	**0.011**
Psychoticism	−0.13	−0.85	0.397	−0.26	−1.54	0.128	−0.22	−1.25	0.214	0.12	0.73	0.465	−0.09	−0.53	0.599

PCL-5, PTSD Checklist for DSM-5; NACM, Negative Alterations in Cognition and Mood; AAR, Alterations in Arousal and Reactivity.

Bold values indicate significant effects.

**Figure 2:**
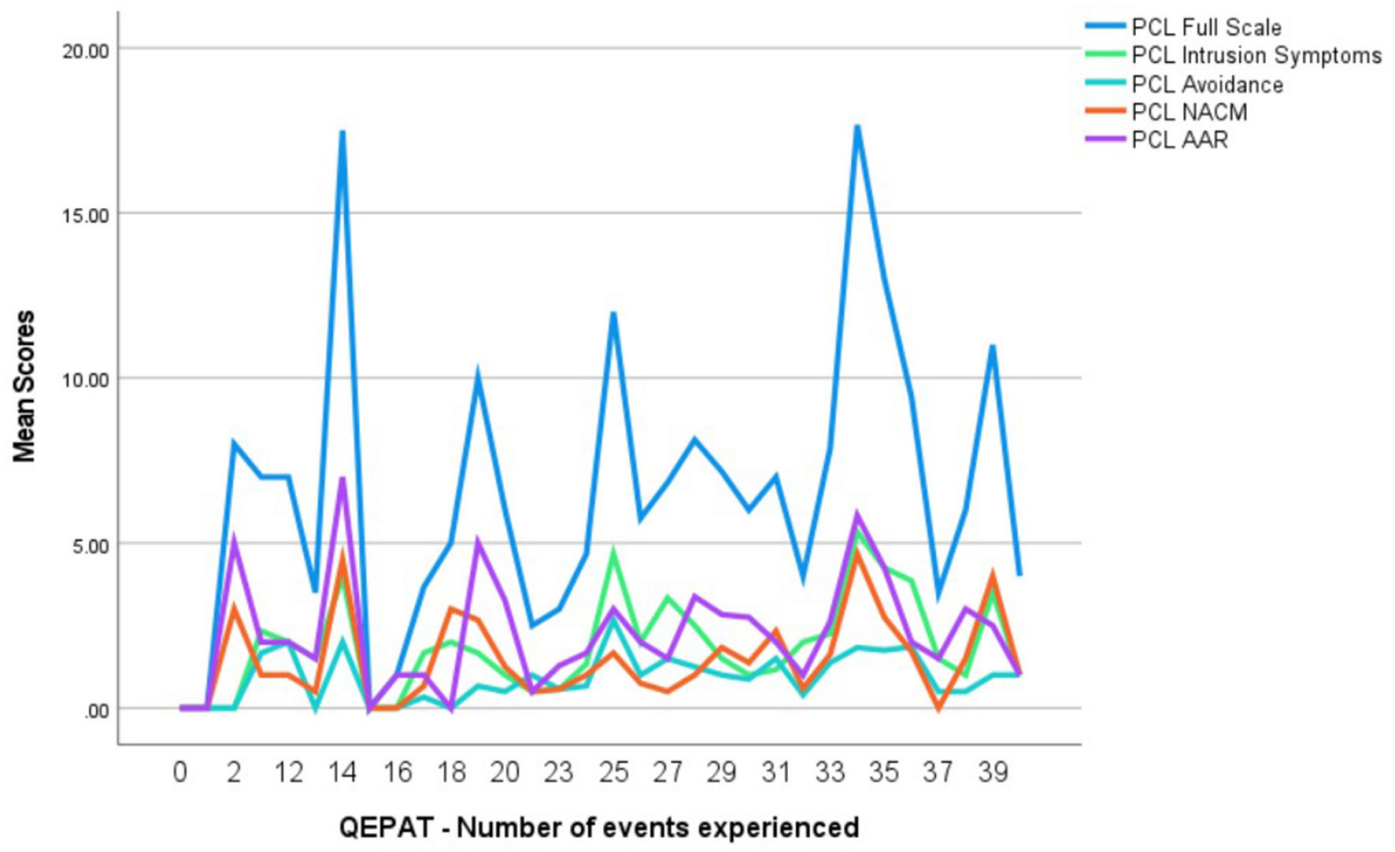
PCL-5 scale and subscale scores per number of adverse events (QEPAT). QEPAT, *Questionário de Exposição e Perturbação dos Acontecimentos Traumáticos*; PCL-5, PTSD Checklist for DSM-5; NACM, Negative Alterations in Cognition and Mood; AAR, Alterations in Arousal and Reactivity.

A simple linear regression was additionally run to test if experiencing a higher number of adverse events, as measured by QEPAT, would lead to higher post-traumatic stress value scores in the PCL-5 (Table 5). The frequency reported for each event was dichotomized to distinguish if the participants had or had not experienced the event. The effect was positive as a higher number of adverse experiences during firefighter service predicted PCL-5 full-scale scores [β = 0.21, *t*(116) = 2.31, *p* = 0.023], albeit only contributing to explaining 4% of the PCL-5 values [*R*^2^ = 0.04, *F*_(1, 116)_ = 5.32, *p* = 0.023]. Regarding the PCL-5 subscales, the number of events experienced only predicted Intrusion Symptoms, having a small positive effect [β = 0.23, *t*(116) = 2.59, *p* = 0.011] and explaining only 6% of scores [*R*^2^ = 0.06, *F*_(1, 116)_ = 6.71, *p* = 0.011] (Table 5). An outline of the PCL-5 full-scale and subscale scores per number of adverse events experienced (according to QEPAT) can be found in Figure 2.

**Table 5:** Simple regression of QEPAT number of events experienced on PCL-5 scale and subscales.

**QEPAT**	**PCL-5 full scale**				**PCL-5 subscales**														
					**Intrusion Symptoms**				**Avoidance**				**NACM**				**AAR**			
	***R** ^2^*	β	***t***	***p***	***R** ^2^*	β	***t***	***p***	***R** ^2^*	β	***t***	***p***	***R** ^2^*	β	***t***	***p***	***R** ^2^*	β	***t***	***p***
Number of events	**0.04**	**0.21**	**2.31**	**0.023**	**0.06**	**0.23**	**2.59**	**0.011**	0.03	0.18	1.96	0.052	0.02	0.15	1.66	0.100	0.01	0.11	1.24	0.219

QEPAT, Questionário de Exposição e Perturbação dos Acontecimentos Traumáticos, a questionnaire related to the exposure and disturbance of traumatic events; PCL-5, PTSD Checklist for DSM-5; NACM, Negative Alterations in Cognition and Mood; AAR, Alterations in Arousal and Reactivity. Bold values indicate significant effects.

The participants were then asked what they considered to be the most significant event they had experienced. The extent to which the participants considered that event to be traumatic predicted PCL-5 full-scale scores [β = 0.38, *t*(116) = 4.47, *p* < 0.001] and accounted for 15% of the total variance [*R*^2^ = *0.1*5, *F*_(1, 116)_ = 19.94, *p* < 0.001]. Being a victim or a witness of the event did not influence PCL-5 full-scale scores [*d* = −0.09, *t*(116) = −0.43, *p* = 0.666].

## 4. Discussion

We have studied a large sample of Portuguese firefighters, after a catastrophic year of deadly fires, from corporations of the District of Coimbra, which mainly consisted of young adult male firefighters, of a single status, with a medium level of education, with a large number (~13.8) years of service and hours (~31.8) of activity per week, and, for the most part, partaking in a voluntary regimen. This question is particularly relevant as we could anticipate that volunteers might present a higher level of vulnerability.

Findings from Stanley et al. (2017) evidenced that volunteers reported greater psychiatric symptoms than career firefighters, which could be justified by greater difficulties accessing mental health treatments related to structural barriers. Some explanations may be raised, namely, the exhaustion caused by the need to conciliate the two types of work (civil and volunteer), the transition between shift hours (mostly during the day off or immediately before or after the work day) and, consequently, sleep deprivation, and less systematic recruitment and training programs. This could all result in a lesser sense of coherence in relation to their firefighting work due to their focus on this activity being reduced compared to their full-time peers (Schnell et al., 2020). The authors define the concept of sense of coherence as the belief that the internal and external environment are predictable, meaningful, and manageable, and it is associated with feelings of preparedness for forthcoming situations. It is possible that similar mechanisms are at play regarding seasonally employed firefighters, who may carry a greater risk of developing PTSD than professional firefighters (Psarros et al., 2008). In an additional interesting conclusion, Dean et al. (2003) stated that more than identifying the nature of firefighter linkage (in this study, professional firefighters reported more psychological distress than the auxiliary group), “psychological distress from the attendance at traumatic events, resulted from a complex interaction of variables such as age, the number of firefighting and non-firefighting traumatic events attended, and length of firefighting service” (Dean et al., 2003). Future works should explore these questions in order to prevent and intervene at earlier stages and identify risk and protective factors in firefighters' work, personal, and family life. This topic is crucial, and the literature has been addressing this issue. For example, in his review, Weisæth (1998) identified pre-, peri-, and post-traumatic factors (protective and risky) as predictors of PTSD. A later review (Tortella-Feliu et al., 2019) emphasized the relevance of these factors to knowledge. They identified 130 potential risk factors, of which 57 were associated with PTSD.

Firefighters regularly face unpredictable events, are exposed continually to several typologies of risk (e.g., physical and psychological), and repeatedly experience a plurality of critical events. In our study, firefighters experienced a mean of ~28 adverse events, and all the events enumerated in the QEPAT were experienced by all of the sample (i.e., none of the events listed in the questionnaire were classified as “never” experienced). One firefighter from the PIT team disclosed having undergone every event (the list contains 43 potentially traumatic/adverse events directly related to firefighting work). These results were in line with data collected by Carvalho and Maia (2009b), who found that 296 Portuguese firefighters from volunteer's corporations in the North of Portugal had experienced a mean of 24 different events (from a list of 40 events in a previous version of QEPAT) since the beginning of their activity. The same number of events was experienced by 736 Chilean volunteer firefighters using the same tool (Spanish version, by N. Ramos and A. Maia in 2015) with 37 items (Ramos, 2019). Our sample reported that the most disturbing event was witnessing a colleague's death or severe injury, and the most frequent experience in active work was where property and goods were at risk of burning. Interestingly, the Chilean sample stated exactly the same pattern of experiences (Ramos, 2019). Due to these idiosyncrasies, one would expect a higher propensity of developing post-traumatic stress symptoms among firefighter workers. Evidence has previously been found that supports this claim (e.g., Berger et al., 2012; Langtry et al., 2021). Similar results of PTSD prevalence in firefighters were found in the present study (prevalence of 8.6%, considering all firefighters who completed the survey (*n* = 139) compared to other Portuguese firefighter samples (e.g., 8% in Marcelino and Gonçalves, 2012; 12% in Pinto et al., 2015; 5.9% in Mesquita, 2018). However, only the latter used the PCL-5 measure. It should be noted that these studies were conducted prior to the 2017 wildfires that ravaged the country and that we found similar values in the prevalence of possible PTSD diagnosis in our study to that found in Mesquita (2018) (to the best of our knowledge, the last study to date published on the prevalence of PTSD in Portuguese firefighters using the same measure—the PCL-5). This is indicative that although these 2017 wildfires were of great magnitude and consequences (San-Miguel-Ayanz et al., 2020), when compared to that found in samples collected prior to 2017, the prevalence of PTSD symptoms in this study (8.6%) appears to not have changed in the following year, which suggests that cumulative adverse events are more important than isolated episodes. However, it was found that the higher the number of reported events during the activity, the higher the severity of PTSD.

When comparing findings of PTSD prevalence, one should take notice of the instruments and procedures used as much heterogeneity is found between studies regarding their methodology, leading to inconsistent prevalence rates (Becker et al., 2022). These differences could bias results and lead to incongruences between findings, thus misrepresenting the true prevalence of PTSD in firefighters. Even though, in our study, the number of participants who fulfilled PTSD criteria for possible PTSD was relatively small (i.e., only 9.2% of participants surpassed either the PCL-5 threshold, fulfilled the DSM-5 criteria, or both in the final sample; *n* = 130), and although it does not represent the majority of firefighters, it is still well above the general population and preponderant enough to warrant specific considerations in order to reflect the pertinence of timely interventions in this field.

One aspect that differentiates those who fulfilled the PTSD criteria from those who did not is how traumatic they considered the most significant event they experienced as a firefighter. More than half of those with PTSD considered the event to have been extremely traumatic, revealing that they were aware of the severity of the incident. Despite that, only one-fourth indicated having had psychological repercussions as a consequence of the event. It should be noted that participants were instructed to respond to the PCL-5 instrument with regard to the most traumatic incident experienced, and so, PTSD scores were indicative of the long-term impact the event had on them. In this regard, there appears to be a possible discrepancy between how traumatic the participants considered the event to be, the psychological impact they considered it had on them, and the actual long-term effects of the traumatic episode. This suggests that these processes may be quite important in this regard.

Several explanations can be put forth for this discrepancy. It is possible that the firefighters sought immediate psychological support from colleagues after the traumatic event and thus did not consider that they had psychological repercussions as a result, even though PTSD symptomatology suggests otherwise. This hypothesis is in agreement with Tamrakar et al. (2020), who found that firefighters rely on informal support from colleagues as an integral avenue of help seeking. It is possible that this acts as a protective factor and that PTSD prevalence would be higher if no support network was established. Another possible alternative explanation is that the firefighters might suppress the psychological and emotional impact of the event. In fact, stigma regarding mental health seems to be prevalent in firefighters (Vujanovic and Tran, 2021) and other first responder populations (Haugen et al., 2017), which could be an obstacle to recognizing psychological distress and subsequently act as a barrier to seeking support. This is particularly concerning considering that out of the 43 events described, the ones identified as being most disturbing were all related to death, injury, or possible injury of colleagues. This evidence highlights the importance of fostering help-seeking behaviors and providing immediate psychological support to firefighters in their corporations when exposed to these situations to prevent the development of PTSD or another psychopathology. Understanding the reasons behind the discrepancy between the reported severity of the event, the perceived psychological repercussions, and PTSD symptoms is key to providing personalized interventions as one or both of the proposed explanations might be at play.

Another explanation could be highlighted, i.e., the resistance that these workers seem to have to trauma exposure (Meyer et al., 2012) and the role that sense of coherence can play in the integration of stressful experiences into the self as a protective factor to develop subsequent PTSD symptoms (Schnell et al., 2020). The sense of coherence can act as a mechanism that is developed to promote the activation of functional coping skills and a psychological preparedness to deal with adverse stressful experiences. These authors also highlighted the indirect influence of resilience in PTSD severity, mediated by the sense of coherence. Their findings were in line with Onyedire et al. (2017), who found that more resilience in Nigerian career firefighters was associated with lower PTSD symptom severity, and a work locus of control (external) predicted PTSD, and with a previous study (Dudek and Koniarek, 2000), which found higher levels of PTSD associated with a lower sense of coherence in firefighters. It is probable, then, that the specific coping mechanisms used immediately after the traumatic event might contribute to increasing or reducing the probability of developing PTSD, as pointed out previously by Dionísio et al. (2022). Future research should address these questions.

Another consideration in our sample is that firefighters who surpassed the PTSD threshold reported also experiencing a greater number of disturbing events than those who did not fulfill the PTSD criteria, which could also contribute to an aggravation of PTSD symptoms.

Another factor that contributed to an increase in PTSD symptoms, in the subsample not signaled for PTSD, was global psychopathology, specifically paranoid ideation, hostility, depression, anxiety, and phobic anxiety symptoms. These results are in part consistent with those of Asmundson and Stapleton (2008), who found that depressive symptoms and anxiety sensitivity predicted PTSD symptom severity of re-experiencing in police officers. Although anxiety and anxiety sensitivity are distinct concepts (for a meta-analysis, see Olatunji and Wolitzky-Taylor, 2009), these are known to be related and could help explain the effect we found on PTSD. The authors additionally concluded that the number of reported traumas predicted PTSD symptom severity (Asmundson and Stapleton, 2008), which is in line with our finding that a higher number of adverse experiences as a firefighter explained PTSD symptoms, albeit a small proportion (4%). On the other hand, while the authors found a positive relationship between depressive symptoms and PTSD, in our study, depression had a negative effect. This unexpected result is contrary to the consensus in the literature regarding the comorbidity between depression and PTSD in firefighters (for a review, see, for example, Salleh et al., 2020). It is possible that this was due to this subsample being specifically composed of participants who did not fulfill the PTSD diagnosis criteria, either by the PCL-5 total score or by DSM-5 rule, and that depressive symptomatology could be independent and not developed as a consequence of trauma exposure by firefighters. Future longitudinal studies could explore if depressive symptoms exist prior to the development of PTSD symptoms or if this comorbidity is a result of adverse event exposure.

An additional caveat should be noted regarding the positive effect (albeit small) of overall psychopathology on PTSD, obtained by the influence of BSI global indexes on PCL-5 scores, and both positive and negative effects of dimensions of psychopathology. It is plausible that the underlying model behaves differently for firefighters who have PTSD and could result in a distinct pattern of responses from participants. Future studies should seek to explore these particular differences with a larger sample of firefighters with possible PTSD diagnoses.

Particularly related to levels of psychopathology and emotional distress, 13.8% of our sample demonstrated expressive values (but with a prevalence of 14.4% when considering all 139 firefighters who completed the survey). This result was in accordance with the existing literature (e.g., Teoh et al., 2019) and close to studies with Portuguese firefighters (Carvalho and Maia, 2009a; Pinto et al., 2015) that demonstrated 17 and 19%, respectively, using the same tool (BSI). Particularly, paranoid ideation, interpersonal sensitivity, depression, hostility, psychoticism, and phobic anxiety scales were the dimensions of the BSI in which more participants (*n* > 20, in each subscale) surpassed the cutoffs^1^. Our results are in line with the literature (e.g., Wagner et al., 2010; Vujanovic and Tran, 2021; Van Hasselt et al., 2022), drawing attention to mental health problems among firefighters.

The profile of psychopathology was partly differentiated in this sample of firefighters. As expected, in our sample, participants with indicators of probable PTSD scored higher mean values than the subsample with subthreshold values of PTSD. Analyzing the normative sample values (BSI) from the general population (Canavarro, 2007), the former subgroup (*n* = 12) presented values of paranoid ideation and the number of positive symptoms (PST index) higher than *M* + 1.5 *Sd* and values for depression and the General Severity Index (GSI) close to this threshold. Conversely, most firefighters (a group with subthreshold values of PTSD) reported normative mean values in the BSI.

More particularly, in our study, the 12 firefighters with probable PTSD seem to have more pronounced mean levels of depression and hostility in contrast to participants without probable PTSD (still, paranoid ideation was the dimension with a higher score for both groups). These results are not surprising since Sindhu et al. (2020) postulated that rates of PTSD and depression in firefighters and other rescue workforces are higher than in the general population, and their study with 100 Indian firefighters found higher levels of substance issues, anger, and sleep problems, followed immediately by depression. Similar results evidencing depressive symptoms among firefighters were found across studies, for example, in Australian (e.g., Harvey et al., 2016), Chinese (Sun et al., 2020), Greek (Psarros et al., 2018), and U.S. samples (Stanley et al., 2017). Although our results are in line with international studies, we found a national study conducted with 312 Portuguese firefighters from the countryside, which revealed normal values of stress, anxiety, and depression and elevated rates of happiness (Afonso et al., 2019).

Regarding hostility, Wagner et al. (2016) suggested that an increased amount of this attribute may be indicative of lower mental health symptoms in firefighters. In addition, Heinrichs et al. (2005) found hostility as a high-risk factor for developing PTSD and psychopathology; moreover, greater scores on hostility and low self-efficacy at baseline (after basic training, prior to trauma exposure) accounted for 42% of the variance in PTSD symptoms after 2 years of professional firefighting service.

The role that personality could play in individuals suffering from PTSD has been previously explored (e.g., Miller, 2003; Psarros et al., 2018; Salleh et al., 2020). In line with the above results, in our study, paranoid ideation and hostility subscales predicted the most variance of PCL-5 scores. Interestingly, these results are in line with the case study analyzed in Becker et al.'s (2022) study as the two Portuguese firefighters studied (male and female, volunteer and career firefighters, respectively) scored higher in those two dimensions of the BSI at baseline. Furthermore, as cited by Sindhu et al. (2020), in 1990, Mitchell evidenced 12 characteristics of personality among emergency service workers (including firefighters) that can contribute to cumulative stress, for example, a need to be in control, obsessive perfectionism, and other compulsive behaviors. Psarros et al. (2018) found that the perception of fear of imminent death during critical firefighting operations and neuroticism may precipitate the development of PTSD in these workers. Future studies should further explore the relation between individuals' traits of personality prior to and after trauma, firefighter duties, PTSD, and the association between the resulting presence of individual mechanisms of response.

Another noteworthy finding in the larger representative sample of firefighters who did not have PTSD was regarding the event they reported as being the most traumatic. The extent to which they considered that event to be traumatic explained 15% of the variance of PTSD measures, which, although a relatively small proportion, can be indicative that these firefighters recognize the impact the event itself had on them. Attention should additionally be brought to the type of events the firefighters are exposed to, in line with Marmar et al.'s (1996) findings. A systematic review of the literature (Wagner et al., 2021b) found that while the impact of large-scale events is relatively consistent across studies, the effect of routine incident exposure on the development of PTSD symptoms showed cross-national differences. Future studies could explore if these differences are found in the QEPAT in firefighters with PTSD as this instrument provides a comprehensive list of both routine and large-scale events firefighters could be exposed to.

One last unexpected result obtained in this study was that even though the participants did not experience a certain event, some still considered a potential occurrence of that event as disturbing. This could be because imagining a hypothetical occurrence, or hearing about it from someone else, would present a disturbing thought in itself and allow them to picture how disturbed they would be when confronted by such an event. This relates to the notion of secondary traumatic stress, defined by Figley (1995, p. 7) as “the natural consequent behaviors and emotions resulting from knowing about a traumatizing event experienced by a significant other—the stress resulting from helping or wanting to help a traumatized or suffering person,” recently found to be present in firefighters in Ankara, Turkey (Bastug et al., 2019). This notion should be explored further in future investigations as it is central to understanding firefighters' needs and developing cross-sectional interventions.

In our sample, the firefighters who surpassed the PTSD threshold experienced a large number of disturbing events, compared to the subsample with subthreshold values of PTSD. This result could explain an aggravation of PTSD symptoms. Both in our and other samples, there are participants who are exclusively professional and those who are exclusively voluntary while also holding other civil occupations, or hybrid members. Future studies might address this question, exploring the differences between voluntary, full-time, and retired firefighters.

An important aspect to consider when interpreting results from this study is that 153 participants did not complete the whole survey, resulting in a dropout rate of 54.1%, which could result in self-sampling bias in the final sample. However, due to the nature and aim of this study (an online survey with a relatively large sample), a more in-depth evaluation (e.g., using a structured interview) was not possible.

Future studies should consider expanding this action research work to a more general sample of firefighters. Thus, future studies could include new tools applied to study firefighter activities and stress responses in controlled environments, namely, virtual reality scenarios, which could also be used to train decision-making processes and stress management strategies.

Additionally, considering the demanding firefighting activity, it could be relevant to study other significant variables for the mental health of these workers, including dissociative peritraumatic experiences, exposition to trauma-related events on an expanded scale, i.e., not just duty activities but also the personal context and its relation with coping strategies related to stress management and cognitive decision-making, and including neuroimagiology data. It might be relevant to include family context indicators, such as childhood adversity and its impact on PTSD indicators and a firefighter's life. The research action nature of the study was achieved as it allowed to characterize this firefighter sample, and a therapeutic response was made available for participants at the CPTTP.

## 5. Conclusion

Due to the idiosyncrasy of their activity, firefighters are continually exposed to adversity and potential traumatic and stress-related events. In the present study, we evaluated the presence and prevalence of psychopathology and PTSD symptoms in a sample of firefighters collected after the fatal wildfires of 2017. The research action nature of the study was achieved as it allowed to characterize this firefighter sample, and a therapeutic response was made available at the CPTTP. We found a similar proportion of firefighters who were signaled for possible psychopathology (13.8%) or fulfilled the PTSD criteria (9.2%) in the final sample (*n* = 130) to that found previously in other Portuguese and international samples. The participants' scores were higher in the paranoid ideation, interpersonal sensitivity, depression, hostility, psychoticism, and phobic anxiety BSI dimensions, which is in line with existing literature. Risk and protective factors for firefighters' mental health were scrutinized as part of a more in-depth look at the vulnerability of these workers.

Additionally, global psychopathology, and specifically paranoid ideation, hostility, depression, anxiety, and phobic anxiety, also contributed to an increase in PTSD symptoms in the majority of firefighters in our sample (i.e., the subgroup who did not fulfill the PTSD criteria). Moreover, experiencing possibly traumatic events as a firefighter also influenced PTSD symptomatology in this subgroup. Both the number of adverse experiences and the perceived severity of the most traumatic event had an impact on PTSD symptom severity, with the latter having the greater effect. This finding is concordant with existing literature on the topic.

However, it should be noted that these conclusions were found for the subsample that did not fulfill the PTSD criteria (*n* = 118), and it is possible that the underlying model structure differs when considering a representative sample of firefighters with PTSD diagnosis. Even though the group that fulfilled the PTSD criteria in this study was too small for comparisons and reliable hypothesis testing (*n* = 12), it is noteworthy that more than half of the participants in this group considered the most traumatic event reported as being extremely traumatic, indicating that they were aware of the severity of the incident. Nevertheless, they indicated not having had psychological repercussions due to the event. The reason for this discrepancy should be further explored in future studies.

## Data availability statement

The raw data supporting the conclusions of this article will be made available by the authors, without undue reservation.

## Ethics statement

The studies involving humans were approved by the local Ethics Committee of the Administração Regional de Saúde do Centro, ARS Centro. The procedures of the study were in accordance with the World Medical Association's Declaration of Helsinki. The studies were conducted in accordance with the local legislation and institutional requirements. The participants provided their written informed consent to participate in this study.

## Author contributions

JO: Data curation, Investigation, Methodology, Formal analysis, Writing—original draft. JD: Data curation, Formal Analysis, Investigation, Methodology, Writing—original draft. ID: Data curation, Formal analysis, Investigation, Methodology, Validation, Writing—review and editing. SC: Investigation, Validation, Writing—review and editing, Methodology. VR: Conceptualization, Methodology, Supervision, Validation, Writing—review and editing. AM: Investigation, Validation, Writing—review and editing, Conceptualization. JR: Investigation, Validation, Writing—review and editing, Conceptualization, Funding acquisition, Supervision. MC-B: Conceptualization, Investigation, Validation, Writing—review and editing, Data curation, Methodology, Project administration, Supervision.

## Footnotes

^1^These thresholds are only informative and were calculated to provide the comparison with the PSDI index within the scope of this work.
