# Supplementary material for: Mental health and post-traumatic stress disorder in firefighters: an integrated analysis from an action research study
Source: Front Psychol. 2023 Oct 27;14:1259388. doi: 10.3389/fpsyg.2023.1259388 (PMC10642162; doi:10.3389/fpsyg.2023.1259388)
Supplement: Supplementary file 1 [file Data_Sheet_1.pdf]

Supplementary Material

Mental health and Post-Traumatic Stress Disorder in firefighters: an integrated analysis from an action-research study

Joana Oliveira†, Joana Aires Dias†, Isabel Catarina Duarte, Salomé Caldeira, António Reis Marques, Vítor Rodrigues, João Redondo[#] and Miguel Castelo-Branco[#,*]

† these authors contributed equally to this work and share first authorship
# these authors shared equal senior autorship

*** Correspondence:** Corresponding Author: mcbranco@fmed.uc.pt

1 Supplementary Figures and Tables

1.1 Supplementary Tables

Characteristics	*f*	Valid %	N
Sociodemographic			
Age (*M*=35.6, *Sd*=10.1, *Md*=36.0, range=19-58)			130
Sex			130
Female	37	28.5	
Male	90	69.2	
Non Disclosed	3	2.3	
Nationality			130
Portuguese	130	100.0	
Education			130
Primary (1º, 2º, 3º, 4º)	2	1.5	
Primary (5º, 6º)	6	4.6	
Primary (7º, 8º, 9º)	26	20.0	
Secondary (10º, 11º, 12º)	58	44.6	
Technical course (not at university)	9	6.9	
Higher education (university)	29	22.3	
Location of employment			130
Predominantly urban	7	5.4	
Predominantly rural	50	38.5	
Mixed urban and rural	73	56.2	
Marital status			130
Single	61	46.9	
Married	46	35.4	
Non-marital Union	19	14.6	
Divorced	4	3.1	

Years together (if married or in non-marital union) (*M*=13.4, *Sd*=9.3, *Md*=12.0, range=1-38)			65
Number of children			130
0	68	52.3	
1	34	26.2	
2	23	17.7	
3	3	2.3	
4	2	1.5	
Age of children			
First (*M*=13.9, *Sd*=9.4, *Md*=12.0, range=0-39)			62
Second (*M*=12.2, *Sd*=7.3, *Md*=12.0, range=1-26)			28
Third (*M*=8.0, *Sd*=7.1, *Md*=6.0, range=1-16)			5
Fourth(*M*=15.5, *Sd*=3.5, *Md*=15.5, range=13-18)			2
Consumption habits			
Alcohol			130
Drinks alcohol	52	40.0	
Does not drink alcohol	75	57.7	
Chose to not respond	3	2.3	
Number of drinks per day			
Beer (*M*=1.5, *Sd*=1.1, *Md*=1.0, range=0-5)			52
Wine (*M*=0.8, *Sd*=1.1, *Md*=0.0, range=0-4)			52
Whiskey (*M*=0.0, *Sd*=0.1, *Md*=0.0, range=0-1)			52
Think they have a drinking problem			130
No	130	100.0	
Yes	0	0.0	
Family thinks they have a drinking problem			130
No	128	98.5	
Yes	2	1.5	
Coffee			130
Drinks coffee	119	91.5	
Does not drink coffee	11	8.5	
Number of coffee cups per day (*M*=2.9, *Sd*=1.3, *Md*=3.0, range=1-8)			119
Smoking			130
Smokes regularly	48	36.9	
Does not smoke regularly	81	62.3	
Chose to not respond	1	0.8	
Number of cigarettes smoked per day (*M*=11.4. *Sd*=5.7, *Md*=10.0, range=2-20)			48
Frequency of medication consumption for anxiety, depression, sleep disorders, etc.			130
Never	102	78.5	
Rarely	14	10.8	
Sometimes	5	3.8	
Regularly	2	1.5	
Daily	7	5.4	
Professional Conditions and Status			
Employment status			130
Unemployed	14	10.8	
Employed	102	78.5	

Student	10	7.7	
Retired	1	0.8	
Other employment status	3	2.3	
Activity sector			130
Primary (agriculture)	1	0.8	
Secondary (service providers)	106	81.5	
Tertiary (industry)	23	17.7	
Firefighting Activity			
Years in service (*M*=13.9, *Sd*=9.8, *Md*=12.0, range=1-40)			130
Work regimen			130
Professional	14	10.8	
Voluntary	82	63.1	
Professional and voluntary	34	26.2	
Firefighting occupation			48
Member of the Permanent Intervention Team (PIT)	11	22.9	
Commander	2	4.2	
Drivers	18	37.5	
Central operator	4	8.3	
Operational assistant	2	4.2	
Other	11	22.9	
Years in specific firefighting occupation (*M*=13.8, *Sd*=9.8, *Md*=12.0, range=1-15)			46
Member of the Permanent Intervention Team (PIT) (*M*=6.4, *Sd*=4.7, *Md*=5.0, range=1-15)			11
Commander (*M*=2.0, *Sd*=0.0, *Md*=2.0, range=2-2)			2
Drivers (*M*=8.7, *Sd*=8.2, *Md*=7.0, range=0-25)			18
Central operator (*M*=6.0, *Sd*=6.1, *Md*=3.0, range=2-13)			3
Operational assistant (*M*=0.5, *Sd*=0.7, *Md*=0.5, range=0-1)			2
Other (*M*=9.6, *Sd*=10.6, *Md*=6.0, range=1-31)			10
Hours worked per week (*M*=31.8, *Sd*=21.8, *Md*=24.0, range=2-84)			130
Member of the Permanent Intervention Team (PIT) (*M*=59.9, *Sd*=8.9, *Md*=60.0, range=50-84)			11
Commander (*M*=42.5, *Sd*=3.5, *Md*=42.5, range=40-45)			2
Drivers (*M*=51.7, *Sd*=14.4, *Md*=50.0, range=10-72)			18
Central operator (*M*=41.3, *Sd*=6.3, *Md*=40.0, range=35-50)			4
Operational Assistant (*M*=53.5, *Sd*=2.1, *Md*=53.5, range=52-55)			2
Other (*M*=57.4, *Sd*=8.9, *Md*=60.0, range=40-70)			11

Supplementary Table 1. *Participants' sociodemographic characteristics, consumption habits, Professional status and Firefighting occupation.*

Variables	1.	2.	3.	4.	5.	6.	7.	8.	9.	10.	11.	12.	13.	14.	15.
**1.** PSDI	-														
**2.** Somatization	.59**	-													
**3.** Obsession-Compulsion	.73**	.62**	-												
**4.** Interpersonal Sensibility	.69**	.53**	.74**	-											
**5.** Depression	.74**	.60**	.79**	.81**	-										
**6.** Anxiety	.65**	.77**	.73**	.67**	.75**	-									
**7.** Hostility	.59**	.57**	.66**	.64**	.70**	.69**	-								
**8.** Phobic Anxiety	.50**	.53**	.59**	.63**	.64**	.67**	.57**	-							
**9.** Paranoid Ideation	.71**	.52**	.66**	.82**	.71**	.68**	.62**	.59**	-						
**10.** Psychoticism	.66**	.51**	.76**	.79**	.81**	.72**	.72**	.61**	.71**	-					
**11.** PCL-5 Total	.33**	.26**	.37**	.46**	.33**	.41**	.49**	.23*	.53**	.39**	-				
**12.** Intrusion Symptoms	.25**	.18*	.24*	.30**	.26**	.26**	.42**	.07	.37**	.24**	.77*	-			
**13.** Avoidance	.25**	.10	.17	.32**	.17	.21*	.29**	.10	.38**	.20*	.70**	.62**	-		
**14.** NACM	.26**	.22*	.36**	.45**	.31**	.43*	.42**	.30**	.47**	.43**	.82**	.39**	.39**	-	
**15.** AAR	.28**	.27*	.36**	.38**	.28**	.38**	.38**	.24*	.46**	.33**	.86**	.45**	.42**	.75**	-

**p*<.05, ***p*<.001

Supplementary Table 2. *Pearson correlation matrix between BSI and PCL-5 scores for non-PTSD subsample.*

Variables	1.	2.	3.	4.	5.	6.	7.	8.	9.	10.	11.	12.	13.	14.	15.
**1.** PSDI	-														
**2.** Somatization	.77*	-													
**3.** Obsession-Compulsion	.87**	.83**	-												
**4.** Interpersonal Sensibility	.84**	.65*	.85**	-											
**5.** Depression	.92**	.64*	.82*	.76*	-										
**6.** Anxiety	.83**	.92**	.93**	.81*	.78*	-									
**7.** Hostility	.63*	.43	.58*	.58*	.68*	.52	-								
**8.** Phobic Anxiety	.63*	.75*	.86**	.66*	.61*	.84**	.38	-							
**9.** Paranoid Ideation	.66*	.71*	.71*	.73*	.53	.73*	.10	.67*	-						
**10.** Psychoticism	.68*	.52	.70*	.81*	.75*	.67*	.56	.61*	.66*	-					
**11.** PCL-5 Total	.52	.56	.40	.22	.61*	.47	.71**	.39	.13	.39	-				
**12.** Intrusion Symptoms	.41	.61*	.43	.32	.31	.50	.61*	.57	.19	.25	.69*	-			
**13.** Avoidance	.09	-.13	-.26	-.07	.18	-.19	.15	-.47	-.14	.19	.22	-.10	-		
**14.** NACM	.16	.24	.17	-.05	.39	.19	.50	.14	-.10	.29	.79*	.20	.16	-	
**15.** AAR	.65*	.54	.57	.36	.66*	.55	.39	.51	.41	.19	.60*	.33	-.27	.38	-

**p*<.05, ***p*<.001

Supplementary Table 3. *Pearson correlation matrix between BSI and PCL-5 scores for PTSD subsample.*
